# The detection, biological function, and liquid biopsy application of extracellular vesicle-associated DNA

**DOI:** 10.1186/s40364-024-00661-2

**Published:** 2024-10-14

**Authors:** Shan Guo, Xin Wang, Danni Shan, Yu Xiao, Lingao Ju, Yi Zhang, Gang Wang, Kaiyu Qian

**Affiliations:** 1 Department of Biological Repositories, Human Genetic Resources Preservation Center of Hubei Province, Zhongnan Hospital of Wuhan University, Wuhan, China; 2 Center for Disease Control and Prevention of Hubei Province, Wuhan, China; 3 Department of Urology, Hubei Key Laboratory of Urological Diseases, Zhongnan Hospital of Wuhan University, Wuhan, China; 4 Human Genetic Resources Preservation Center, Wuhan University, Wuhan, China; 5 Euler Technology, ZGC Life Sciences Park, Beijing, China; 6 Center for Quantitative Biology, School of Life Sciences, Peking University, Beijing, China; 7 Wuhan Research Center for Infectious Diseases and Cancer, Chinese Academy of Medical Sciences, Wuhan, China

**Keywords:** Extracellular vesicles, DNA, Detection, Biological function, Liquid biopsy

## Abstract

Cell-derived extracellular vesicles (EVs), which carry diverse biomolecules such as nucleic acids, proteins, metabolites, and lipids reflecting their cell of origin, are released under both physiological and pathological conditions. EVs have been demonstrated to mediate cell-to-cell communication and serve as biomarkers. EV-associated DNA (EV-DNA) comprises genomic and mitochondrial DNA (i.e., gDNA and mtDNA) fragments. Some studies have revealed that EV-DNA can represent the full nuclear genome and mitochondrial genome of parental cells. Furthermore, DNA fragments loaded into EVs are stable and can be transferred to recipient cells to regulate their biological functions. In this review, we summarized and discussed EV-DNA research advances with an emphasis on EV-DNA detection at the population-EV and single-EV levels, gene transfer-associated biological functions, and clinical applications as biomarkers for disease liquid biopsy. We hope that this review will provide potential directions or guidance for future EV-DNA investigations.

## Background

**Fig. 1:**
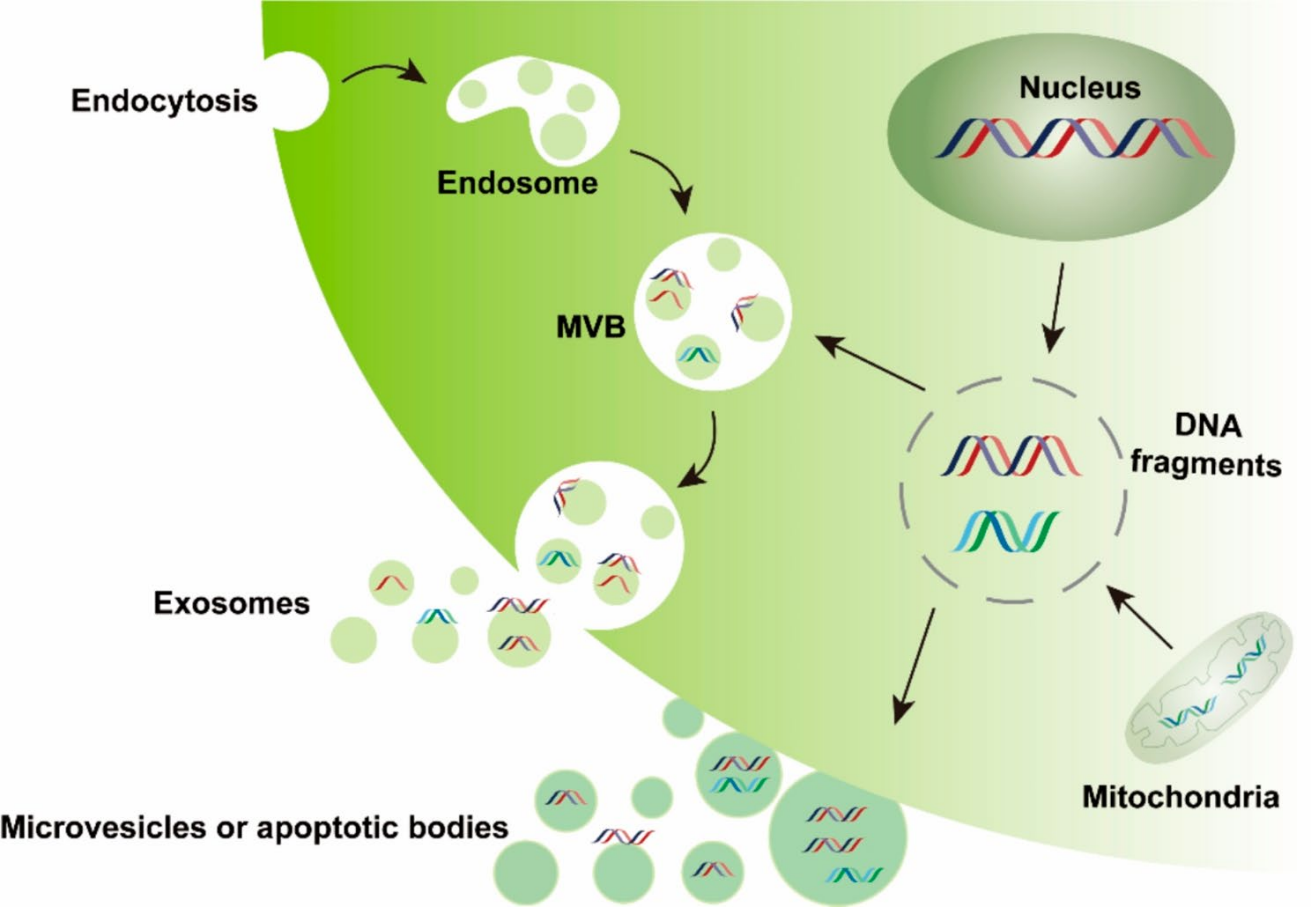
Schematic illustration of heterogeneous EVs carrying diverse DNA fragments. EVs can be released by plasma budding or form in the multivesicular body (MVB) followed by release through the exocytosis pathway

EVs are particles that are released from cells and delimited by a lipid bilayer membrane [1, 2]. EVs can either pinch off the surface of the plasma membrane via outward budding (e.g., microvesicles with a diameter of 100–1000 nm or apoptotic bodies with a diameter of 1000–5000 nm) or can be generated inside multivesicular endosomes or multivesicular bodies (MVBs) via double invagination of the plasma membrane and then are released to the extracellular space through the exocytosis pathway (i.e., exosomes with a diameter of 50–150 nm) (Fig. 1) [3, 4]. These EVs enclose many constituents of parent cells, including nucleic acids, proteins, and metabolites, and display a wide range of sizes. EVs are implicated in cell-to-cell communication, allowing cells to exchange components and influencing various pathophysiological processes in both parent and recipient cells [5–10].

EVs are present in various tissues (e.g., the brain, melanoma, adipose, and liver) [11–16] and body fluids (e.g., plasma, urine, breast milk, ascites, saliva, cerebrospinal fluid, and bile) [17–21]. The lipid membrane of EVs can protect their cargoes against degradation, particularly for nucleic acids [22–25]. Compared with EV-associated RNA (EV-RNA), DNA molecules were later found within EVs and have been less investigated. However, EV-DNA has attracted increasing interest, and corresponding advances are ongoing. Single-strand DNA (ssDNA), double-strand DNA (dsDNA) or chromatin DNA, and mtDNA, have been detected inside and/or outside EVs enriched from in vitro cultured cells and biofluids such as plasma [26–32], serum [33, 34], urine [35], gastric juice [36], saliva [37], pleural effusion [38], and lymphatic drainage [39]. In this review, we summarize and discuss advances in EV-DNA-associated detection, biological function, and liquid biopsy applications.

EVs are a highly heterogeneous population manifesting in their size, content (cargo), source (cell of origin), and functional impact on recipient cells [3]. There is a lack of defined nomenclature for EV populations. In addition to biogenesis-related terms such as exosome, microvesicle, and ectosome, operational terms, such as small EVs (sEVs) and large EVs, are commonly used to denote EV subtypes in published papers [1]. sEVs are usually obtained with a diameter of generally < 200 nm after separation via methods such as differential ultracentrifugation (dUC) or filtration [1, 2]. Compared with large EVs, sEVs are widely prepared as starting materials and are studied more in terms of EV-DNA characterization, functions, and applications. As such, this review pays more attention to DNA fragments derived from a mixed population of sEVs without further demonstration of their intracellular origin. In addition, as there is no strict consensus on upper and lower size cut-offs [1], the term EVs is used in the manuscript except that sEVs need to be highlighted.

## EV-DNA detection

**Fig. 2:**
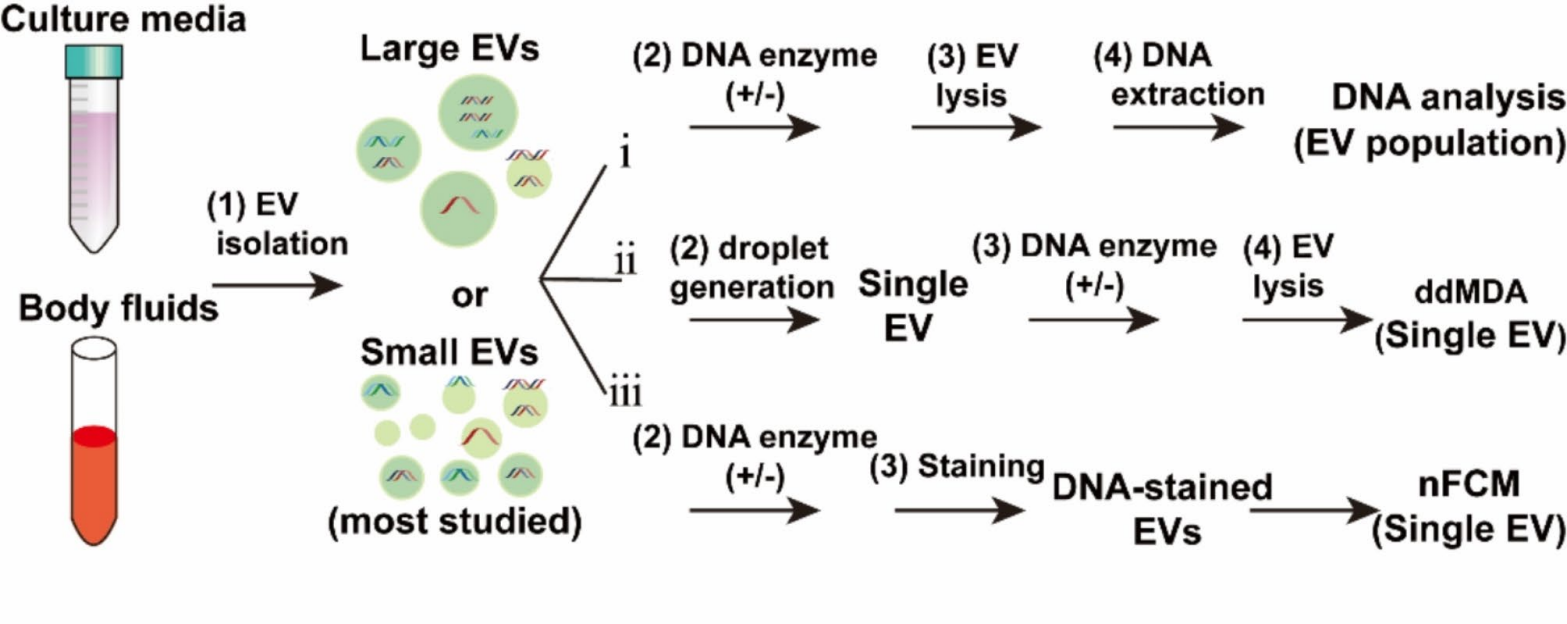
Schematic workflows of EV-DNA detection at the population-EV and single-EV levels. In general, EVs are first isolated from culture media or body fluids, followed by DNA enzyme digestion, EV lysis, DNA extraction, and DNA analysis. When DNA analysis is performed via nanoflow cytometry (nFCM), EV lysis and DNA extraction are not needed, but DNA staining is needed. When DNA cargoes are profiled in a single EV via hydrogel-based droplet digital multiple displacement amplification (ddMDA), EV lysis is still needed, while DNA extraction is not needed

Typically, EV-DNA detection requires three steps: EV isolation, DNA extraction, and DNA characterization (Fig. 2). On the basis of physical characteristics (such as size and density) and biochemical properties (e.g., surface protein markers), a variety of approaches have been developed to isolate EVs; these approaches have been summarized in many reviews [40–46] and are not introduced here. As current studies have performed DNA analyses on EV populations and single EVs, we reviewed EV-DNA detection at the population-vesicle and single-vesicle levels.

### EV-DNA detection at the population-vesicle level

Cells can release a large number of EVs in culture media or biofluids (e.g., approximately 10^9^/mL in peripheral blood) [47, 48]. However, EVs are relatively small in size, and the amount of fragmented gDNA or mtDNA is limited [49]. Thus, EV isolation and DNA extraction methods are concerned. Currently, dUC is widely used to isolate or concentrate EVs from culture media or body fluids. For EV-DNA isolation, various commercial kits that are applied to extract DNA from cells, blood or tissues have also been used to isolate DNA from EVs, such as the QIAamp Micro Kit (Qiagen), the QIAamp DNA Mini Kit (Qiagen), the DNeasy Blood and Tissue Kit (Qiagen), the MagAttract HMW DNA Kit (Qiagen), and the GenElute Mammalian Genomic DNA Miniprep Kit (Sigma‒Aldrich) [26, 27, 30–35]. These DNA isolation kits use a silica membrane or magnetic beads to selectively bind DNA and do not require toxic phenol‒chloroform extraction or ethanol precipitation. In addition, few EV-DNA isolation kits that integrate EV and DNA isolation have also been developed on the market; for example, the EV-DNA isolation kits of Duolaimi Biotechnology Co., Ltd. (Wuhan, China) & GeMExo Biotech Corp. (http://www.dlmbiotech.com) were used. Unfortunately, few comparative studies have been performed on existing EV and DNA isolation methods to explore the optimal procedure for EV-DNA isolation from culture media, body fluids, or tissues.

For DNA analysis, DNA enzymes, including DNase I, S1 nuclease, and dsDNase, are used to determine which forms of DNA fragments (e.g., ssDNA or dsDNA) are loaded into EVs [50–54]. To determine the location of DNA fragments inside or outside EVs, isolated EVs are treated with or without DNA enzymes before EV lysis. For quantitation, the EV-DNA concentration can be determined via ultraviolet absorbance-based Thermo Scientific™ NanoDrop™ Spectrophotometer (with a detection range of 2‒12,000 ng/µL) or a fluorescence-based fluorometer [28]. Comparatively, fluorometric quantitation with a DNA-binding fluorescent dye is more sensitive and specific for the nucleic acid of interest. However, before testing, samples for fluorometric quantitation need to be processed with kits such as the Invitrogen™ Qubit™ dsDNA HS Assay Kit (with a detection range of 0.1‒120 ng), the Invitrogen™ Quant-iT™ PicoGreen™ dsDNA Assay Kit (with a detection range of 50 pg‒2 µg), and the Promega™ QuantiFluor™ dsDNA System (with a detection range of 0.01‒200 ng, https://www.promega.com.cn*).* In addition, the length of EV-DNA fragments can be determined via agarose gel electrophoresis with a DNA ladder/marker, Agilent Bioanalyzer 2100 instrument, or Agilent 4200 TapeStation instrument. Sequencing results revealed that EV-DNA fragments isolated from EV populations span all chromosomes and mitochondrial DNA [51, 55].

Although DNA fragments have been detected from EVs, there is no consensus on EV-DNA parameters including location, form, concentration, and size. It seems that large EVs or EV surfaces carry complex DNA fragments with greater weights [52, 56, 57]. In addition, factors that can cause DNA damage and cellular damage or stress increase the amount of DNA fragments loaded into EVs from culture media or body fluids [58–65]. Takahashi et al. reported that abnormal accumulation of harmful DNA in the cytosol could activate the DNA damage response and trigger an aberrant immune response via the cGAS (cylic GMP-AMP synthase)-STING (a stimulator of interferon genes) signaling pathway [66]. Thus, EV-mediated DNA excretion from cells is presumed to be helpful for maintaining cellular homeostasis. Similarly, EV-mediated mtDNA release may be necessary to maintain subcellular mitochondrial homeostasis [67]. However, these assumptions need to be proven in the future.

Meanwhile, some studies have explored how nuclear DNA and mtDNA fragments are loaded into EVs [68, 69]. In 2019, Yokoi et al. preliminarily explored the mechanism by which nuclear contents are loaded into exosomes [68]. They reported that MVBs and the tetraspanin CD63 biomarker directly interact with the micronuclei (MN), suggesting that gDNA-containing exosomes are likely produced following MN collapse, where their nuclear contents are shuttled into MVBs via tetraspanins [68]. Recently, Zhang et al. reported that the transcription factor FOXM1 interacts with LC3 in the nucleus and transfers specific chromatin DNA fragments, including the DUX4 gene and telomere DNA, to EVs through secretory autography during the lysosome inhibition process [69]. This finding revealed for the first time how chromatin DNA fragments are specified to EVs. With respect to how mtDNA fragments are loaded into EVs, mitochondrial-derived vesicles (MDVs) seem to be involved in this process [70]. MDVs have been proposed as another method for controlling mitochondrial quality in addition to the mitochondrial–lysosome axis [71, 72]. Like MN-mediated DNA fragments packaged into MVBs [68], mtDNA fragments are likely routed to exosomes after MDVs interact with MVBs; however, this hypothesis needs to be proven. In addition, EVs are enriched in lipids such as cholesterol, phospholipids, and sphingolipids [73, 74]. Cytoplasmic DNA is taken up in intraluminal vesicles possibly via its interaction with the lipid raft-like region of the MVB membrane [75].

However, Jeppesen et al. argued that exosomes do not carry DNA or DNA-binding histones [76]. To obtain exosomes, they first used the dUC method to isolate crude sEVs, followed by purification with high-resolution iodixanol density gradient fractionation. Then, immunoaffinity beads targeting exosomal tetraspainins are used to specifically isolate exosomes from other types of sEVs, which fail to collect marker-negative sEV subpopulations [76]. Through the use of the same method for isolating sEVs from fresh human plasma, Lichá et al. reported that 60–75% of the DNA remained on the surface of sEVs and that a portion of the DNA was localized inside the sEVs [52]. In addition, Zhang et al. used asymmetric flow-flow fractionation to identify two sEV subpopulations with diameters of 90–120 nm and 60–80 nm as well as nonmembranous nanoparticles termed ‘exomeres’ (~ 35 nm), and reported that these particles have unique DNA profiles [77]. Altogether, DNA fragments can be detected from bulk EVs; however, the high intrinsic heterogeneity of EV populations may lead to various and even contentious results in terms of EV-DNA features. It is necessary to develop optimal enrichment methods to obtain more homogeneous EV subpopulations for accurate characterization of EV-DNA fragments.

### EV-DNA analysis at the single-vesicle level

Currently, technologies for single EV analysis, such as nanoflow cytometry (nFCM), atomic force microscopy, droplet digital polymerase chain reaction (ddPCR), digital ELISA, and immunofluorescence imaging, have been developed to facilitate in-depth comprehension of various EV subtypes with differential physical properties, molecular compositions, or biological roles [41, 49, 78–82]. However, few studies have been carried out to analyze EV-DNA at the single-EV level thoroughly. On the basis of a laboratory-built nFCM, Liu et al. detected single EVs bearing DNA fragments labeled with the membrane-permeable nucleic acid stain SYTO™ 16 (Fig. 2) [83]. This laboratory-built nFCM method can analyze single EVs as small as 40 nm in diameter and single DNA fragments of 200 bp. In addition, in combination with enzymatic treatment, the results revealed that (1) naked DNA or DNA associated with nonvesicular entities was abundantly present in EV samples prepared from cell culture media by dUC; (2) the quantity of EV-DNA in individual EVs exhibited high heterogeneity, and the population of DNA-positive EVs varied from 30 to 80% depending on the cell type; (3) external EV-DNA was mainly localized on relatively small EVs (e.g., < 100 nm for the HCT-15 cell line), and the secretion of external DNA-positive EVs could be significantly reduced by exosome secretion pathway inhibition; (4) internal EV-DNA was mainly packaged inside the lumen of relatively large EVs (e.g., 80–200 nm for the HCT-15 cell line); (5) dsDNA was the predominant form of both the external and internal EV-DNA; (6) histones (H3) were not found in EVs, and EV-DNA was not associated with histone proteins; and (7) genotoxic drugs induced an increased release of DNA-positive EVs and the number of both external DNA-positive EVs and internal DNA-positive EVs as well as the DNA content.

Additionally, Jiao et al. developed a hydrogel-based droplet digital multiple displacement amplification (ddMDA) approach for the comprehensive analysis of EV-DNA at the single-EV level [84]. EV samples were prepared via dUC, and then, single EVs were dispersed in thousands of cross-linked poly(ethylene glycol) hydrogel droplets and lysed for DNA amplification and identification (Fig. 2). The results revealed that (1) 5 − 40% of EVs were associated with DNA, and significant differences existed not only between normal and tumor cells but also between tumor cells treated with anticancer drugs and untreated cells; (2) compared with EVs with a mean diameter of 109.7 ± 59.1 nm, EVs with a mean diameter of 170.4 ± 95.6 nm presented a greater proportion of DNA-containing EVs and a more substantial presence of intraluminal DNA; (3) these DNA-containing EVs carry multiple DNA fragments on average; and (4) both dsDNA and ssDNA were detected at the single-EV level. These two studies used EVs isolated from in vitro culture media as the starting material for EV-DNA analysis at the single-EV level. The features of EV-DNA fragments derived from body fluids or tissues remain to be examined at the single-EV level.

Taken together, current methods for EV-DNA detection at either the population-EV or single-EV level require isolation of EVs from culture media or body fluids in advance (Fig. 2). Hence, the EV isolation method is critical and determines which type of EV is used for subsequent DNA analysis. It is unknown whether the characteristics of isolated EV-DNA fragments are consistent with those of the original culture media and body fluids. The optimal EV isolation method for accurate characterization of EV-DNA should be explored.

## Biological functions of EV-DNA

**Fig. 3:**
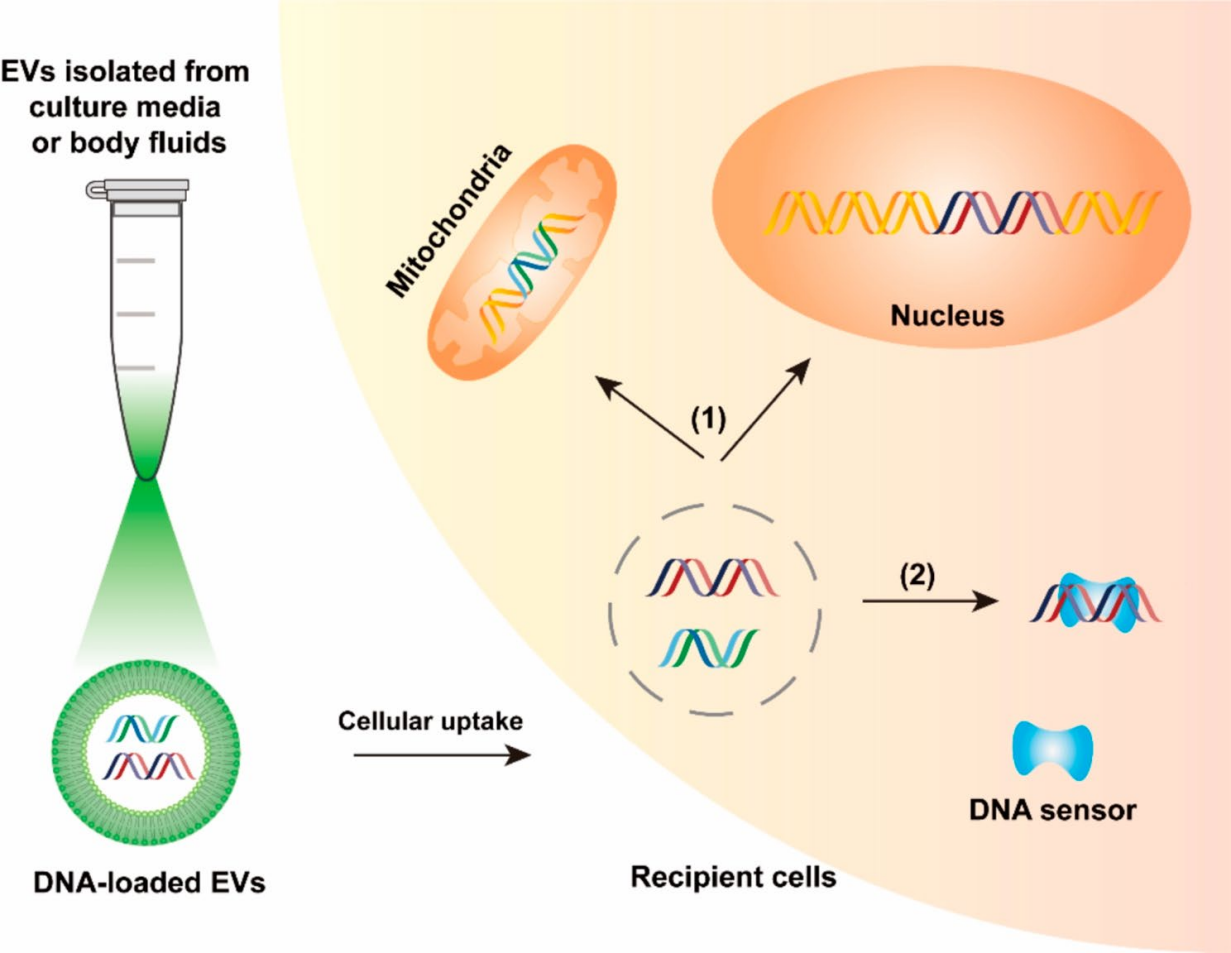
Schematic overview of DNA-containing EVs acting on recipient cells. Heterogeneous-source DNA-containing EVs can be transferred to recipient cells and transform or affect their biological responses: (1) EV-DNA may translocate to the recipient cell nucleus and/or mitochondria and be integrated into the host genome, resulting in changes in the gene expression and/or phenotype of recipient cells; (2) EV-DNA may activate cytosolic DNA sensors of recipient cells, triggering the innate immune or inflammatory response

EVs released from donor cells can be taken up and convey their molecular cargoes, including DNA, to recipient cells via receptor‒ligand interactions, endocytosis and/or phagocytosis or even membrane fusion, thereby exerting effects on recipient cells. Accumulating evidence has revealed that EV-DNA can be transferred to recipient cells through horizontal transmission and even vertical transmission from parents to offspring. After uptake, on the one hand, EV-DNA can offer additional gene materials to recipient cells, leading to changes in gene transcription, protein translation, and/or phenotype (Fig. 3). On the other hand, EV-DNA serves as a signal molecule to activate cytoplasmic DNA-sensing pathways (e.g., cGAS-STING and the AIM2 inflammasome) and drive the immune or inflammatory response in recipient cells (Fig. 3).

### EV-DNA serves as an additional gene material contributing to changes in gene expression and/or the phenotype of recipient cells

In 2012, Waldenström et al. reported for the first time that DNA-stained (with acridine orange) EVs derived from the culture media of cardiomyocytes were transferred to target fibroblasts and could be seen in the fibroblast cytosol and even in the nuclei [85]. However, it is not clear whether EV-DNA transfer into recipient cells is functional. Since then, increasing evidence indicates that EVs contain functional genes or chromosomal DNA fragments and telomeres and transfer them to recipient cells, resulting in changes in gene expression and/or phenotypes [86–94]. Cai et al. reported that an endogenous promoter of the AT1 (angiotensin II type 1) receptor, NF-kB, could be recruited to the transferred DNAs in the nucleus and increase the transcription of the AT1 receptor in recipient HEK293 cells [86]. In addition, unique BCR/ABL hybrid gene-containing EVs derived from human chronic myelogenous leukemia K562 cells were found to be transportable to HEK293 cells or neutrophils, resulting in the expression of the BCR/ABL hybrid gene mRNA and protein in the recipient cells [86]. K562 cell-derived EVs were injected into the tail vein of Sprague‒Dawley rats and immunodeficient NOD/SCID mice, and some characteristics of chronic myeloid leukemia, such as fever, thinning, splenomegaly, and neutrophilia, but reduced neutrophil phagocytic activity were observed [87]. Lanna et al. also reported that EV-mediated telomere transfer from antigen-presenting cells (APCs) to T cells (primarily naïve and central memory cells) during initial synaptic contact with APCs could elongate the telomeres of T cells and make them stemlike and/or central long-lived memory cells, conferring long-lasting immune protection [88].

Additionally, EVs bearing tumor-associated oncogene DNA or mutated gene DNA were found to be able to drive a protumorigenic phenotype in normal recipient cells [89–92]. EVs derived from patients with dedifferentiated liposarcoma (DDLPS) or DDLPS cell lines carry MDM2 oncogene DNA. These EVs can be transferred to preadipocytes, leading to impaired p53 activity in preadipocytes and increased proliferation, migration, and production of matrix metalloproteinase 2 [89]. Domenis et al. showed that mutated dsDNA (TP53 c.818G > A and KRAS c.35G > T) in EVs derived from the human colon cancer cell line SW480 could be actively transcribed in normal CCD841-CoN colon epithelial and THLE-2 hepatic cells, thereby transforming normal cells and modifying their phenotypes, such as proliferation and migration [90, 91].

With respect to cancer therapy, studies have shown that EV-mediated mtDNA transfer helps cancer cells acquire resistance [55, 93]. Hormonal therapy can induce oxidative phosphorylation-deficient breast cancer cells. However, the impaired metabolism in cancer cells could be rescued via the transfer of mtDNA-laden EVs derived from cancer-associated fibroblasts, promoting escape from metabolic quiescence and hormonal therapy-resistant metastatic breast cancer [55]. Additionally, EVs from chemoresistant triple-negative breast cancer cells can transfer mtDNA to sensitive cancer cells by increasing mtDNA levels with mutations in the mtND4 gene (which is responsible for tumorigenesis), thus leading to acquired chemoresistance [93].

More recently, Bolumar et al. discovered that maternal endometrial EVs could mediate vertical DNA transmission to preimplantation embryos and demonstrated that the internalization of EV-derived nuclear-encoded (n)DNA/mtDNA by trophoblast cells of murine embryos was associated with a reduction in mitochondrial respiration and ATP production [94]. This finding suggested that EV-mediated vertical transmission of maternal DNA was associated with altered embryo bioenergetics during the periconception period. Taken together, these results indicate that EVs bearing DNA fragments from donor cell nuclear DNA or cytoplasmic mtDNA can be internalized and incorporated into the genome of recipient cells, resulting in corresponding functional or phenotypic changes.

### EV-DNA serves as a signal molecule triggering innate immunity in immune cells

The innate immune response is the first line of defense against infection by bacterial and fungal pathogens. Abnormal DNA in the cytosol of cells can be sensed by DNA sensors such as toll-like receptor 9, cGAS, and STING and absent in melanoma 2 (AIM2), which mediates type I interferon (IFN) production and inflammasome activation [95–99]. With respect to tumor treatment, Kitai et al. reported that cancer cells treated with the antitumor drug topotecan (TPT) secreted DNA-loaded EVs, which could activate dendritic cells (DCs) via a STING-dependent pathway and produce inflammatory cytokines [100]. In vivo, TPT administration inhibited tumor growth in tumor-bearing mice, which was accompanied by the infiltration of activated DCs and CD8^+^ T cells [100]. For radiotherapy, mouse breast carcinoma cells treated with 8 Gy X 3 released dsDNA-containing EVs [101]. Likewise, EV-DNA was shown to stimulate DC upregulation of costimulatory molecules and STING-dependent activation of IFN [101]. In vivo, irradiated tumor cell-derived EVs were found to elicit tumor-specific CD8^+^ T-cell responses and significantly better protect mice from tumor development than EVs from untreated tumor cells in a prophylactic vaccination experiment [101]. Furthermore, Lv et al. reported that nonionizing ultraviolet radiation and ionizing radiation (X-ray and Boron neutron capture therapy) had different effects on EV-DNA fragments derived from tumor cells [102]. Boron neutron capture therapy induced more DNA fragments in tumor cell-derived EVs. These DNA-loaded EVs were also shown to activate the DNA-sensing pathway in DCs and enhance their functions, including antigen presentation and migration capacity [102]. After these EV-educated DCs are vaccinated, the effector T cells significantly expand and infiltrate into tumors [102]. These results suggest that EV-DNA derived from treated cancer cells can activate immune cells and elicit protective antitumor immunity.

In addition, EV-DNA was found to be involved in intestinal immune and inflammatory responses [103–105]. Lian et al. reported that chemotherapeutic irinotecan (CPT-11) can induce the packaging of a large amount of gDNA and mtDNA into the EVs of intestinal cells, which can activate the AIM2 inflammasome in innate immune cells (e.g., macrophages and DCs), promote the secretion of the mature cytokines IL-1β and IL-18, and cause intestinal toxicity [103]. In addition to chemotherapy-induced intestinal immune-dyfunctional response, EV-DNA has also been shown to participate in the development of inflammatory bowel disease (IBD), such as Crohn’s disease [104, 105]. A high content of EV-DNA, including mtDNA and nuclear DNA fragments, was detected in the plasma or colon lavage of murine colitis and Crohn’s disease patients and was positively correlated with disease activity [104]. Zhao et al. discovered that EVs from the plasma of active human Crohn’s disease and LPS-damaged colon epithelial cells could trigger STING activation and increase inflammation in macrophages, whereas the effect disappeared after removal of EV-DNA via sonication and dsDNase to digest dsDNA in or out of the EVs [104]. In IBD patients, gut microbiota-derived EV-DNA was also shown to induce barrier function damage and inflammatory responses in epithelial cells via the cGAS/STING pathway [105].

Additionally, EV-mediated DNA transfer has been shown to play an important role in pathogen infection progression [106–108]. EVs from malaria parasite (*Plasmodium falciparum*)-infected red blood cells contain parasite gDNA, which can be internalized by monocyte cells and stimulate STING-TBK1-IRF3-dependent gene induction [108]. Additionally, Torralba et al. reported that the interaction of T cells with antigen-bearing DCs could initiate the antipathogenic programs of DCs [109]. T-cell-derived EVs contain gDNA and mtDNA, which can stimulate the cGAS/STING pathway and induce the expression of IRF3-dependent interferon-regulated genes in DCs [109]. T-cell EV-treated DCs were more resistant to subsequent viral infections. These findings indicate that the interaction of T cells with DCs has physiological consequences for DC functions. T cells prime DCs through EV-mediated DNA transfer, suggesting a specific role for antigen-dependent contacts in conferring protection to DCs against pathogen infection.

Taken together, these findings indicate that EV-DNA transfer from donor cells to recipient cells has physiological and pathological significance. However, the detailed mechanisms underlying EV-DNA uptake are poorly understood. Recipient cell responses to EVs have been shown to rely on the donor cell source and dose [110]. The EV dose had a more significant effect than the cell source; however, EV cell source-specific responses were observed at low doses [110]. In addition, as EVs convey complex molecular cargoes to neighboring or distant cells, some new phenotypic and molecular responses in recipient cells may be attributed to other regulatory molecules in EVs or synergistic effects of all cargoes in EVs. It is strongly necessary to deplete the DNA cargo within EVs to determine EV-DNA-induced function.

## Liquid biopsy application of EV-DNA

**Fig. 4:**
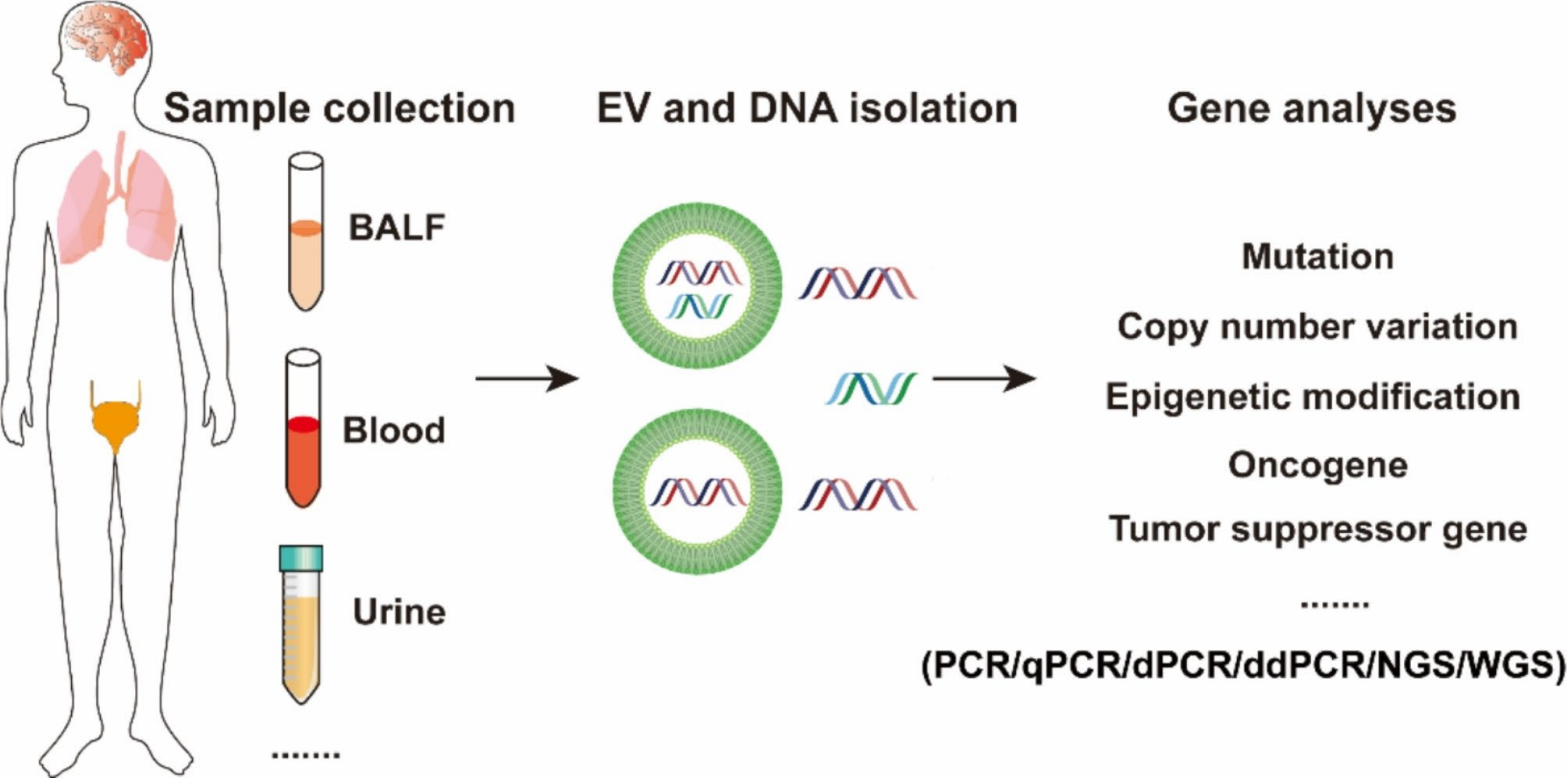
Schematic illustration of EV-DNA-based gene analyses for liquid biopsy. EV-DNA isolated from body fluids can be used for various gene analyses (including mutation, copy number variation, epigenetic modification, etc.) with the help of diverse PCR and sequencing techniques

Liquid biopsy is defined as the sampling and analysis of components (e.g., circulating tumor cells, circulating cell-free DNA (cfDNA), circulating tumor DNA (ctDNA), circulating tumor RNA, and exosomes present in body fluids such as blood, urine, and saliva [111–113]. Compared with tissue biopsy, liquid biopsy is minimally invasive or even noninvasive, which facilitates its routine clinical use in patients for disease diagnosis and dynamic monitoring of disease progression and treatment response. For EVs, the membrane bilayer structure has a protective effect on the encapsulated cargoes against degradation. DNA in serum EVs was reported to be stable for 1 week at 4 °C, 1 day at room temperature, and after repeated freeze‒thaw cycles (less than three times) [24]. Clearly, DNA fragments inside EVs are more stable than cfDNA. cfDNA was reported to have a short half-life of 2–2.5 h [114]. Furthermore, EVs can reflect their cell of origin and are associated with the physiological and pathological status of the body. Given these advantages, EV-DNA has been considered an alternative resource for gene detection and screening EV-DNA-based markers for disease diagnosis and monitoring (Fig. 4) [115–122]. Many studies have explored the potential of EV-DNA-based liquid biopsy for the treatment of cancers such as lung cancer, pancreatic cancer, urinary cancer, and nervous system tumors [123–152].

### Lung cancer

Current studies have shown that samples collected from lung cancer patients, including blood plasma, pleural effusion, and bronchoalveolar lavage fluid (BALF), contain EV-DNA with detectable epidermal growth factor receptor (EGFR) mutations (e.g., exon 19 deletion, p.L858R, p.T790M) (Table 1) [38, 123, 124, 126, 127]. In both plasma and BALF samples from non-small cell lung cancer (NSCLC) patients, EV-DNA-based analysis showed greater agreement with conventional tissue biopsy than did cfDNA-based liquid biopsy [123]. In particular, the test results of the BALF samples were significantly greater than the results of the plasma samples for both ctDNA and EV-DNA, indicating that proximal biofluids better represented the tumor status [123]. Furthermore, the use of EV-DNA from BALF samples was more effective than tissue biopsy for detecting the p.T790M mutation in patients who developed resistance to epidermal growth factor receptor-tyrosine kinase inhibitors (EGFR-TKIs) [123]. In addition to mutation analysis, Batochir et al. identified specific methylation patterns in the EV-DNA of lung cancer BALF and reported that combinations of seven epigenetic biomarkers (including HOXA9, HOXD3, PCDH1, NID2, NPTX2, RASSF1A, and SFRP2) were capable of discriminating between lung cancer and benign lung diseases suspected of lung malignancy [125].

**Table 1:** EV-NA- and cfDNA-based gene analyses in lung cancer

Cancer type	Sample type	EV collection	EV-DNA extraction	EV-NA extraction	cfDNA extraction	EGFR mutation	Gene analysis	Comparison outcome	Reference
NSCLC	Blood plasma and BALF	dUC	High-Pure PCR Template Preparation Kit (Roche Diagnostics, Mannheim, Germany)		High-Pure PCR Template Preparation Kit (Roche Diagnostics, Mannheim, Germany)	Exon 19 deletion, p.L858R, p.T790M	PNAClamp™ EGFR Mutation Detection Kit (Panagene, Daejeon, Korea)	EV-DNA outperform cfDNA	[123]
Pulmonary adenocarcinoma	Pleural effusions	dUC	High-Pure PCR Template Preparation Kit		High-Pure PCR Template Preparation Kit	Exon 19 deletion, p.L858R, T790M	PNAClamp™ EGFR Mutation Detection Kit (Panagene, Daejeon, Korea)	EV-DNA outperform cfDNA	[126]
Stage IV lung adenocarcinoma	Pleural effusion	dUC		ExoLution Plus Isolation Kit (Exosome Diagnostics)	QIAamp Circulating Nucleic Acid Kit (Qiagen)	416 cancer-relevant genes	Targeted NGS	Comparable	[38]
NSCLC	Blood plasma	ExoQuick™ (System Biosciences, Mountain View, CA, USA)	MagMAX Cell-Free DNA Isolation Kit (Thermo Fisher Scientifc)	MagMAX™ Total Nucleic Acid Isolation Kit (Thermo Fisher Scientifc)	MagMAX CellFree DNA Isolation Kit (Thermo Fisher Scientifc, Waltham, MA, USA)	Exon 19 deletion, p.L858R, p.T790M	ddPCR	EV-NA outperform cfDNA and EV-DNA	[127]

With respect to the specimen of pleural effusion, the results revealed that (1) EV-DNA- and cfDNA-based EGFR genotyping for patients who were either EGFR-TKI naïve without EGFR-TKI treatment or who acquired resistance to EGFR-TKI also showed good agreement with tissue biopsy; (2) the detection rate of p.T790M using EV-DNA was more efficient than that using cell blocks or cfDNA [126]. In 18 patients who acquired resistance to EGFR-TKIs with the p.T790M mutation, EGFR genotyping via EV-DNA, cfDNA, and cell blocks detected this mutation in 13, 11, and 3 patients, respectively [126]. Targeted next-generation sequencing (NGS) results revealed that the genetic profiles of EV-DNA and cfDNA isolated from pleural effusions of stage IV lung adenocarcinoma patients were comparable, except for copy number variations (CNVs), which presented lower similarities between these two samples [38].

In addition to comparisons between EV-DNA and cfDNA, EV-associated nucleic acids (EV-NAs), including DNA and RNA, were extracted from the blood plasma samples of NSCLC patients, and their clinical utility was explored. ddPCR-based EGFR mutation test results showed that short-length EV-NAs (~ 200 bp) contained more detectable tumor-derived nucleic acids than EV-DNA (~ 200 bp in length or full length) or cfDNA. Short-length EV-NAs and cfDNA generally showed good concordance with the tissue EGFR results. The sensitivity of liquid biopsy using EV-NAs was greater than that of liquid biopsy using cfDNA [127]. Overall, EV-DNA, especially proximal biofluid-derived EV-DNA, is promising as an alternative source for lung cancer liquid biopsy.

### Pancreatic cancer

Human blood samples are often collected from pancreatic cancer patients for the detection of cancer-related mutations in EV-DNA [128–133] (Table 2). The concentration of EV-DNA in serum was reported to be greater than that in plasma; however, the mutant allele fraction (MAF) of KRAS in EV-DNA in serum was lower [129]. In addition, the levels of tumor-derived mutant KRAS DNA were highest in association with large EVs and sEVs early and with sEVs and soluble proteins late in disease progression, indicating that sEVs were the most enriched in tumor-derived DNA throughout disease progression [130].

**Table 2:** EV-DNA- and cfDNA-based gene analyses in pancreatic cancer

Sample origin	Sample type	EV collection	EV-DNA extraction	cfDNA extraction	Gene aberration	Gene analysis	Comparison outcome	Reference
Patients with pancreatic ductal adenocarcinoma, chronic pancreatitis and intraductal papillary mucinous neoplasm, and healthy human subjects	Blood serum	dUC	QIAamp DNA Micro Kit (Qiagen)		KRAS^G12D^ and TP53^R273H^ mutations	dPCR		[128]
Patients with I-IV stages of pancreatic cancer	Blood serum/plasma	ExoEasy Maxi Kit (Qiagen)/ dUC	QIAamp DNA Micro Kit (Qiagen)		KRAS mutants at codons 12/13	ddPCR		[129]
Patients with pancreatic cancers of known mutant KRAS G12 genotype	Blood plasma	dUC/ Size-exclusion chromatography (Izon SP1)	Phenol: Chloroform: Isoamyl alcohol		KRAS ^G12V^ KRAS ^G12D^	dPCR		[130]
Patients with advanced pancreatic cancer	Blood plasma	dUC/qEV size exclusion chromatography/ Total Exosome Isolation precipitation	DNeasy Blood & Tissue Kit (Qiagen)	QIAamp Circulating Nucleic Acid kit (Qiagen)	KRAS mutants at codons 12/13	ddPCR	comparable	[131]
Patients with metastatic disease, localized disease, pancreatic cysts and nonneoplastic pancreatic disease	Blood plasma	dUC	QIAamp Circulating Nucleic Acid Kit (Qiagen)	QIAamp Circulating Nucleic Acid Kit (Qiagen)	KRAS mutants at codons 12/13	ddPCR	EV-DNA outperform cfDNA	[132]
Patients with localized, locally advanced, and metastatic pancreatic ductal adenocarcinoma and healthy controls	Blood plasma	dUC	MagAttract High Molecular Weight DNA kit (Qiagen)	QIAmp Circulating Nucleic Acid Kit (Qiagen)	KRAS mutants at codons 12/13	ddPCR	EV-DNA outperform cfDNA	[133]

ddPCR-based gene testing revealed that mutant KRAS EV-DNA was present in 7.4%, 66.7%, 80%, and 85% of age-matched healthy controls and localized, locally advanced, and metastatic pancreatic ductal adenocarcinoma patients, respectively [133]. Similarly, mutant KRAS cfDNA was detected in 14.8%, 45.5%, 30.8%, and 57.9% of these individuals [133]. A greater percentage of pancreatic ductal adenocarcinoma patients presented detectable KRAS mutations in EV-DNA than in cfDNA. In 48 clinically annotated serum samples from pancreatic ductal adenocarcinoma patients, dPCR analyses of EV-DNA revealed the KRAS^G12D^ mutation in 39.6% of patients and the TP53^R273H^ mutation in 4.2% of patients [128]. KRAS^G12D^ and TP53^R273H^ mutations were detected in EV-DNA from intraductal papillary mucinous neoplasm patients and chronic pancreatitis patients. Notably, KRAS mutations were identified in EV-DNA from healthy controls, indicating the need for careful consideration and application of liquid biopsy findings [128].

In addition to EV-DNA detection for the diagnosis of pancreas-associated pathologies, the clinical utility of EV-DNA and cfDNA KRAS MAFs in patients with localized and metastatic pancreatic ductal adenocarcinoma was determined, and the results were compared [132]. Compared with standard readouts, such as imaging and carbohydrate antigen 19 − 9, the dynamics of KRAS mutation detection in circulating nucleic acids, including EV-DNA and cfDNA, could be correlated with disease progression. In 34 patients with potentially resectable tumors, an increase in EV-DNA after neoadjuvant therapy was significantly associated with disease progression, whereas ctDNA did not correlate with outcomes [132]. The concordance rates of KRAS mutations present in surgically resected tissue and detected in liquid biopsy samples were greater than 95%. These findings suggest that EV-DNA-based mutation analyses have great potential for pancreatic cancer diagnosis and monitoring.

### Urinary cancer

Among urinary cancers, body fluids from urothelial bladder carcinoma (UBC), prostate cancer, and renal cell carcinoma have been reported to contain EV-DNA [35, 56, 57, 134, 135] (Table 3). For prostate cancer, large EVs carry most of the tumor DNA in patient blood plasma, whereas negligible amounts of DNA are presented in the sEVs from the same patients [56, 57]. Whole-genome sequencing (WGS) revealed that plasma EV-DNA from patients with metastatic prostate cancer could represent tumor genomic features and reflect genetic aberrations in the cell of origin [134]. With respect to UBC, cfDNA and EV-DNA extracted from urine samples from patients undergoing surgical treatment with somatic mutations and CNV similar to those of tumor tissues were measured [35]. Nonetheless, these findings are preliminary, and more tests are needed to explore the clinical utility of EV-DNA as an alternative biomarker for urinary cancer. In addition, as urine is a specific sample for urinary cancers and can be easily collected in a truly noninvasive manner, urine-derived EV-DNA gene analysis may be more effective for detecting urinary cancers.

**Table 3:** EV-DNA- and cfDNA-based gene analyses in urinary cancer

Cancer type	Sample type	EV collection	EV-DNA extraction	cfDNA extraction	Gene aberration	Gene analysis	Comparison outcome	Reference
UBC	Urine	ExoQuick-TC (System Biosciences, Mountain View, CA)	QIAamp DNA Mini Kit (Qiagen, Valencia, CA)	MagMax Cell-Free DNA Isolation Kit (Thermo Fisher Scientific)	somatic mutations of 9 genes and CNV	Target gene capture sequencing/sWGS	Comparable	[35]
Prostate cancer	Blood plasma	dUC/iodixanol density gradient centrifugation (Optiprep™)	QIAamp DNA Micro Kit (Qiagen)			WGS		[56]
Prostate cancer	Blood plasma	dUC	DNeasy Blood and Tissue Kit (Qiagen)		MLH1, PTEN, and TP53 genes	real time qPCR		[57]
Renal cell carcinoma	Blood plasma	dUC	Slagboom buffer with rproteinase K		mitochondrial genes (such as HV1 and CYB)	qPCR/dPCR/ NGS		[135]

### Nervous system tumors

For nervous system tumors, glioma- and neuroblastoma-derived EV-DNA have been analyzed to identify tumor-associated genes, gene mutations, and gene modifications such as methylation [136–140]. In glioblastoma multiforme, the NANOG and SOX2 genes were detected in EV-DNA isolated from conditioned culture media [136, 137]. Genome-wide methylation profiling of glioblastoma cell-derived EVs revealed that EV-DNA could reflect genome-wide methylation profiles [138]. Rosas‑Alonso et al. detected of O6-methylguanine-DNA methyltransferase methylation in the plasma EV-DNA of glioblastoma patients with a remarkable sensitivity of 85.7%, and suggested EV-DNA-based liquid biopsy for monitoring disease progression in IDH-wild type glioblastoma patients [139]. Regarding neuroblastoma, EVs from blood plasma samples from patients were determined to contain dsDNA [140]. Whole exome sequencing results revealed that such EV-DNA carried tumor-specific genetic mutations, including those occurring on known oncogenes and tumor suppressor genes in neuroblastoma (ALK, CHD5, SHANK2, PHOX2B, TERT, FGFR1, and BRAF), and represented the entire exome [140]. Furthermore, neuroblastoma-derived EV-DNA was useful for identifying variants responsible for acquired resistance, such as mutations in the ALK, TP53, and RAS/MAPK genes that occur in relapsed patients [140].

In addition to the abovementioned tumors, biofluids from other cancers, such as lymphoma [28], pediatric acute myeloid leukemia [30], colorectal cancer [141–143], colon cancer [144], breast cancer [145, 146], gastric cancer [36], oropharyngeal squamous cell carcinoma [147], epithelial ovarian cancer [148], pheochromocytoma [149], paraganglioma [149], melanoma [150], hepatocellular carcinoma [151], and osteosarcoma [152], are sporadically reported having EV-DNA molecules.

More recently, some studies have shown that EV-DNA exists in samples of noncancerous diseases [153, 154]. The copy number of mtDNA in the blood EVs of patients with cardiovascular disease was found to be greater than that in healthy subjects [153]. Song et al. also detected mutations in the plasma EV-DNA of patients with pulmonary nodules and developed a compact 21-gene panel for the differential diagnosis of malignant PN and benign PN [154]. At the NCBI website (http://clinicaltrials.gov*)*, 9 clinical trials have been registered for exploring the role of EV-DNA in cancer screening (NCT06192875), cancer diagnosis (NCT03236675, NCT05854563, NCT04164134, and NCT04742608), occurrence and development of gastric cancer (NCT05956847) and acute respiratory distress syndrome caused by extrapulmonary sepsis (NCT05061212), and treatment monitoring (NCT03228277 and NCT03217266) (Table 4). Among them, only two trials, NCT04164134 and NCT03228277, were completed, but no results were posted. Much more effort is still needed to validate EV-DNA-based biomarkers in independent cohorts or prospective trials and establish EV-DNA-based liquid biopsies for cancer or noncancerous diseases in the future.

**Table 4:** EV-DNA associated clinical trials registered at the NCBI website

ClinicalTrials.gov ID	Official title	Sample type	Status
NCT06192875	A Novel Molecular Approach to Blood DNA Screening for Cancer: Specificity Assessment (The NOMAD Study)	Blood/urine/saliva	Recruiting
NCT03236675	Detection of Either the EML4-ALK Gene Rearrangements or the T790M EGFR Mutation in the Plasma of Advanced NSCLC Patients	Blood plasma	Unknown
NCT05854563	Cough Capture as a Portal into the Lung-ICTR Pilot	Cough	Recruiting
NCT04164134	New Strategies to Detect Cancers in Carriers of Mutations in RB1: Blood Tests Based on Tumor-educated Platelets, or Extracellular Vesicles.	Blood	Completed
NCT04742608	Development of Liquid Biopsy Technologies for Noninvasive Cancer Diagnostics in Patients with Suspicious Thyroid Nodules or Thyroid Cancer	Blood	Suspended
NCT05956847	The Role of Extracellular DNA (ecDNA) in the Occurrence and Development of Gastric Cancer: an Exploratory Multicenter Prospective Observational Clinical Study	Blood	Recruiting
NCT05061212	The Mechanism of Extracellular Vesicles Containing Mitochondrial DNA in acute respiratory distress syndrome (ARDS) Lung Injury Caused by Extrapulmonary Sepsis	Blood plasma	Unknown
NCT03228277	Phase II, Multicenter, Single-arm, Open-label Study to Evaluate the Efficacy of Olmutinib(Olita^®^) in Patients With NSCLC Who Harboring T790M Mutation Confirmed Using DNA Extracted From Extracellular Vesicles in Bronchoalveolar Lavage Fluid	BALF	Completed
NCT03217266	A Phase Ib Trial of Neoadjuvant AMG 232 (KRT-232) Concurrent with Preoperative Radiotherapy in Wild-Type P53 Soft Tissue Sarcoma (STS)	Blood	Active, not recruiting

## Conclusions and outlooks

In summary, attractive advances have been made in the field of EV-DNA research. Nuclear gDNA and/or mtDNA fragments have been discovered in EVs isolated from culture media and various biofluids. Furthermore, EV-DNA has been shown to play diverse roles in multiple physiological and pathological processes and potentially serves as an alternative gene material for disease liquid biopsy. However, technologies that enable the isolation of homogeneous EV subpopulations from either culture media or biofluids are lacking. dUC has been extensively used to isolate and distinguish sEVs from large EVs, but the obtained EVs are still a mixed population that cannot better reflect their biogenesis, cell or tissue origin. It is essential to develop novel approaches capable of separating EV subpopulations from each other. In addition, as there are no standard protocols for EV and DNA isolation, EV-DNA obtained via different approaches may present controversial characteristics. The optimal procedures for EV-DNA isolation should also be investigated to define EV-DNA features well.

In addition to the discovery and validation of DNA within EVs, how EV-DNA is formed and released from donor cells followed by uptake, internalization, and function in recipient cells remains to be further explored to better understand the molecular mechanisms behind EV-DNA functions. For tissue-derived EVs, studies focused on DNA are lacking and should be carried out in the future. With respect to liquid biopsy, DNA inside EVs seems to be more stable than cfDNA without a lipid bilayer coating. More studies should be carried out to investigate the translation potential of EV-DNA for disease diagnosis and monitoring. Notably, disease-specific samples such as BALF for NSCLC, urine for bladder cancer, and bile for hilar cholangiocarcinoma are likely more efficient for liquid biopsy. In addition to mutations, other cancer-associated gene aberrations and epigenetic modifications remain to be comprehensively profiled in EV-DNA from different body fluids. Taken together, continuous research is still needed to comprehensively characterize EV-DNA features, deeply parse EV-DNA functions, and better apply EV-DNA for disease liquid biopsy.

## Data Availability

No datasets were generated or analysed during the current study.

## Abbreviations

EVs: Extracellular vesicles
sEVs: Small EVs
MVBs: Multivesicular bodies
MN: Micronuclei
MDVs: Mitochondria-derived vesicles
EV-DNA: EV-associated DNA
EV-RNA: EV-associated RNA
EV-NAs: EV-associated nucleic acids
ssDNA: Single-strand DNA
dsDNA: Double-strand DNA
gDNA: Genomic DNA
mtDNA: Mitochondrial DNA
cfDNA: Cell-free DNA
ctDNA: Circulating tumor DNA
cGAS: cylic GMP‒AMP synthase
STING: Stimulator of interferon genes
AIM2: Absent in melanoma 2
IFN: Type I interferon
TPT: Topotecan
DCs: Dendritic cells APCs
APCs: antigen-presenting cells
AT1: Angiotensin II type 1
EGFR: Epidermal growth factor receptor
EGFR-TKIs: Epidermal growth factor receptor-tyrosine kinase inhibitors
dUC: Differential ultracentifugation
nFCM: Nanoflow cytometry
ddMDA: Droplet digital multiple displacement amplification
PCR: Polymerase chain reaction
qPCR: Quantitative PCR
dPCR: Digital PCR
ddPCR: Droplet digital PCR
NGS: Next-generation sequencing
WGS: Whole-genome sequencing
sWGS: Shallow WGS
CNVs: Copy number variations
MAF: Mutant allele fraction
DDLPS: Dedifferentiated liposarcoma
IBD: Inflammatory bowel disease
NSCLC: Non-small cell lung cancer
BALF: Bronchoalveolar lavage fluid
UBC: Urothelial bladder carcinoma
PN: Pulmonary nodules
